# Patient classification and attribute assessment based on machine learning techniques in the qualification process for surgical treatment of adrenal tumours

**DOI:** 10.1038/s41598-024-61786-w

**Published:** 2024-05-16

**Authors:** Marta Wielogórska-Partyka, Marcin Adamski, Katarzyna Siewko, Anna Popławska-Kita, Angelika Buczyńska, Piotr Myśliwiec, Adam Jacek Krętowski, Agnieszka Adamska

**Affiliations:** 1 Department of Endocrinology, Diabetology and Internal Medicine, Medical University of Bialystok, Bialystok, Poland; 2 Faculty of Computer Science, Bialystok University of Technology, Wiejska 45A, 15-351, Bialystok, Poland; 3 Department of General and Endocrine Surgery, Medical University of Bialystok, Bialystok, Poland; 4 Clinical Research Centre, Medical University of Bialystok, Bialystok, Poland

**Keywords:** Endocrinology, Mathematics and computing

## Abstract

Adrenal gland incidentaloma is frequently identified through computed tomography and poses a common clinical challenge. Only selected cases require surgical intervention. The primary aim of this study was to compare the effectiveness of selected machine learning (ML) techniques in proper qualifying patients for adrenalectomy and to identify the most accurate algorithm, providing a valuable tool for doctors to simplify their therapeutic decisions. The secondary aim was to assess the significance of attributes for classification accuracy. In total, clinical data were collected from 33 patients who underwent adrenalectomy. Histopathological assessments confirmed the proper selection of 21 patients for surgical intervention according to the guidelines, with accuracy reaching 64%. Statistical analysis showed that Supported Vector Machines (linear) were significantly better than the baseline (*p* < 0.05), with accuracy reaching 91%, and imaging features of the tumour were found to be the most crucial attributes. In summarise, ML methods may be helpful in qualifying patients for adrenalectomy.

## Introduction

### Aim

An adrenal incidentaloma (AI) is an asymptomatic adrenal mass that is recognized incidentally during imaging examinations and is not associated with suspected adrenal pathology^1,2^. Incidental discovery of adrenal masses has increased recently due to wider application and technical improvement of abdominal imaging procedures, with a prevalence of approximately 0.2–6.9% in radiological studies^1,3–5^. A comprehensive hormonal evaluation of newly diagnosed adrenal masses at their initial presentation was recommended by the European Society of Endocrinology in 2016^6^.

Patients should be referred for adrenalectomy with clinically significant hormone excess, radiological findings suspicious for malignancy, signs of local invasion, and when the tumour is greater than 5 cm^6^. Underlying comorbidities, advanced age, and Hispanic ethnicity were associated with more frequent postoperative complications. Therefore, the coexistence of heart failure or respiratory failure should always be considered before qualifying for surgical treatment of adrenal tumours^7^.

The primary objective of this study was to compare several machine learning (ML) techniques in a qualification for adrenalectomy and choose the most accurate algorithm as a valuable adjunct tool for doctors to simplify making therapeutic decisions by using the most innovative and modern methods. To the best of our knowledge, this study is the first attempt to apply ML techniques to qualify for the surgical treatment of AI using both the results of diagnostic tests and computed tomography (CT) image features. Preliminary results of this study were presented in a poster session at the European Congress of Endocrinology^8^.

### Related works

In the literature, most studies apply computer vision techniques to recognize the type of tumour based on CT images^9–16^. In one study, the authors evaluated ML-based texture analysis of unenhanced CT images in differentiating pheochromocytoma from lipid-poor adenoma in adrenal incidentaloma^10^. The textural features were computed using the MaZda software package, and two classification methods were used: multivariable logistic regression (accuracy of 94%) and number of positive features by comparison to cut-off values (accuracy of 85%). The results were encouraging; however, decision classes were unbalanced and the accuracy values were computed on the test set. Therefore, they were biased estimators. In another study, the authors applied a multivariable logistic regression model with 11 selected textural features computed using MaZda software^11^. The cut-off point obtained using the eceiver operating characteristic (ROC) curve applied to the expression obtained from logistic regression resulted in a sensitivity of 93% and 100% specificity. Again, these results were obtained using the same set used to train the model. In another study performed by Li et al., ML models were used to differentiate pheochromocytoma from lipid-poor adenoma based on the radiologist’s description of unenhanced and enhanced CT images^9^. The authors used three classifiers: multivariate logistic regression, SVM and random forest. As a result, two separate models based on multivariable logistic regression were proposed, each using three CT features: M1 with preenhanced CT value, shape, and necrosis/cystic changes (accuracy of 86%) and M2 using only preenhanced CT features: CT value, shape, and homogeneity (accuracy of 83%). Elmohr et al. used the ML algorithm to differentiate large adrenal adenomas from carcinomas on contrast-enhanced computed tomography, and its diagnostic accuracy for carcinomas was higher than that of radiologists^13^. Other studies have evaluated the accuracy of ML-based texture analysis of unenhanced CT images in differentiating lipid-poor adenoma from pheochromocytoma, with performance accuracy ranging from 85 to 89%^10,14^.

The literature also includes papers applying ML techniques to magnetic resonance imaging (MRI) data. An example of such work is a study where the authors utilized logistic regression with the least absolute shrinkage and selection operator (LASSO) to select MRI image features and distinguish between non-functional AI and adrenal Cushing’s syndrome^17^.

In studies involving a large number of features (e.g.: software packages such as MaZdA can calculate several hundred texture parameters for images), dimensionality reduction is required. Techniques commonly used (or combinations of them) are: LASSO with regression^18–21^, elimination of correlated features^9,21^ or those with low intraclass correlation (ICC)^18^, training of classifiers for subsets of features and selection of subsets with the highest classifier accuracy^9^, elimination of features with p-values above the accepted error rate for coefficients in regression models, use of feature discrimination power calculated using the ROC curve for each feature separately^10^.

Artificial neural networks (ANN) are flexible and powerful ML techniques that have evolved from the idea of simulating the human brain, however their successful application usually requires datasets much larger that other classification methods^17–19^.

To improve the quality of patient care, recent studies have been conducted in several different sectors using modern techniques. There are two types of ML-based models: current-condition identification and forward prediction^20^. In Table 1, we have summarized studies concerning the utilization of ML techniques in AI management.

**Table 1:** Summary of studies looking at the application of ML techniques in AI management.

Study	ML task	Sample size	ML classifier	Main findings	Main limitations
Yi et al.^10^	To differentiate between subclinical pheochromocytoma (and lipid-poor adenoma in cases of AI using texture features of unenhanced CT scans	80 patients with lipid-poor adenoma and 29 patients with subclinical s pheochromocytoma	Logistic regression (accuracy of 94%) and number of positive features by comparison to cut-off value (accuracy of 85%)	ML-based quantitative texture analysis on unenhanced CT scans appears to offer a dependable quantitative approach for distinguishing between pheochromocytoma and lipid-poor adenoma in cases of AI	Discrepancy in sample sizes between the two groups. Lack of division of data into training and test datasets. Results for one ML classification method only
Yi et al.^14^	To differentiate between subclinical pheochromocytoma and lipid-poor adrenal adenoma in AI using texture and other parameters of CT images	181 patients with lipid-poor adenoma and 84 patients with subclinical pheochromocytoma	Logistic regression using contrast-enhanced CT (AUC of 0.967), and using pre-enhanced CT (AUC of 0.958)	ML approach for pre-enhanced and enhanced CT images distinguished subclinical pheochromocytoma from lipid-poor adenoma. In particular, a good result for CT without contrast allows to avoid the additional radiation and risk associated with enhanced CT	Discrepancy in sample sizes between the two groups. Results for one ML classification method only
Elmohr et al.^13^	To distinguish large adrenal adenomas and carcinomas using texture features of precontrast and venous CT images and tumour attenuation values	25 patients with adrenocortical adenoma and 29 patients with adrenocortical carcinoma	Logistic regression (accuracy of 82%, texture features and attenuation) and Boruta random forest (accuracy of 76%, texture features only)	CT texture analysis of large adrenal tumours and carcinomas is likely to improve CT evaluation of AI	Highly selective nature of the included adrenal tumours Delayed-phase CT images were not included. Results for one ML classification method only
Liu et al.^9^	To differentiate subclinical pheochromocytoma from lipid-poor adenoma in patients with AI using parameters of pre-enhanced and enhanced CT images analysed by radiologists	183 patients with lipid-poor adenoma and 86 patients with subclinical pheochromocytoma	Logistic regression model (best accuracy of 86%), SVM and Random Forest (lower accuracy than LR, no exact figures were given)	The promising application of CT-based ML models and scoring systems for predicting the histology of AI was demonstrated	Lack of arterial phase and multi-phase scans of CT. Results for one ML classification method only
Maggio et al.^11^	To differentiate between cortisol secreting and non-secreting AI using texture features of CT scans in non-contrast phase	40 patients with functioning and 32 with non-functioning adrenal masses	Logistic regression (sensitivity of 93.75% and a specificity of 100%)	CT texture analysis shows potential as a valuable tool in defining the diagnosis of AIs	Large number of features incorporated into the predictive model. Results for one ML classification method only
Yang et al.^15^	To distinguish between aldosterone-producing adenoma from non-functioning adrenal adenoma using contrast-enhanced CT image features combined with clinical features	68 patients with aldosterone-producing adenoma 60 patients with non-functioning adrenal adenoma	Logistic regression using CT image features (accuracy of 73%) and logistic regression combining CT and clinical features (accuracy of 96%)	Contrast-enhanced CT -based radiomics and clinical radiomics ML model exhibited good diagnostic efficacy in differentiating aldosterone-producing adenoma from non-functioning adrenal adenoma	Only patients with contrast-enhanced CT imaging data were included. Highly selective nature of the included tumours. Results for one ML classification method only
Piskin et al.^21^	To differentiate between nonfunctioning and autonomous cortisol-secreting AI using texture features of unenhanced MRI images	100 patients with adrenal lesions	Logistic regression, best results using MRI image features (AUC of 0.758)	Non-functioning AI and autonomous cortisol-secreting AI can be distinguished with high accuracy on unenhanced MRI Radiomics analysis and the model built using ML algorithms appear to be superior to radiological assessment method	Results for one ML classification method only
Piskin et al.^22^	To differentiate between non-functional adrenal incidentaloma and adrenal Cushing’s syndrome in cases of AI using texture features of MRI	50 patients with AI	Logistic regression (best model AUC 0.994)	The developed MRI-based radiomic scores can yield high area under curves for prediction of adrenal Cushing’s syndrome	The assessment of interobserver reproducibility in feature extraction was not feasible as only one radiologist assessed the images Results for one ML classification method only
Feliciani et al.^16^	To differentiate between pathologically proven adenomas and other adrenal histotypes using texture features of unenhanced CT images	48 patients with 50 adrenal lesions	Four classifiers were used: logistic regression (AUC of 0.96), linear discriminant (AUC of 0.95), linear SVM (AUC of 0.94), decision tree (AUC of 0.91)	The research constructed a radiomic signature based on unenhanced CT scans to categorize lipid-poor adenomas	Lack of control over CT scanner types due to the retrospective nature of the study

## Materials and methods

### Study population

From a database of 264 Caucasian patients with AI, the clinical data of 33 patients older than 18, who met the criteria for surgical treatment according to the guidelines of the Polish Society of Endocrinology, were used in this retrospective, single-center study^23^. Patients had been hospitalized and qualified for an operation in the Department of Endocrinology, Diabetology, and Internal Medicine at the University Clinical Hospital in Białystok between 2017 and 2019. All qualified patients underwent laparoscopic lateral transperitoneal adrenalectomy.

We searched our institutional electronic database and confirmed proper qualifications in 21 of the 33 patients selected for operation according to the obtained results of postoperative histopathological examinations. Definitive diagnoses were established through histopathology, revealing a study group comprising five cases of pheochromocytomas, two cases of ACCs, five cases of Cushing’s syndrome, and nine cases of primary hyperaldosteronisms. The remaining 12 cases consisted of patients with benign, hormonally inactive lesions, for whom surgical intervention was unnecessary. This study complied with the Declaration of Helsinki and was approved by the Ethical Committee of Białystok (no. APK.002.14.2022). Informed consent for study participation was obtained from all enrolled patients.

### Biochemical and radiographic analyses

All patients completed a comprehensive endocrine work-up aimed at studying the hormonal status of AI: aldosterone/renin ratio, 24 h urine collection for metanephrines and normetanephrines, and 1 mg overnight DXM suppression test. Serum cortisol levels after 1 mg DXM > 5 µg/dL confirmed hypercortisolism, whereas serum concentrations of cortisol between 1.9 and 5.0 µg/dL were considered evidence of possible autonomous cortisol secretion. To confirm the diagnosis of CS, the serum concentration of ACTH was measured. The diagnosis of primary aldosteronism was confirmed with a saline infusion test. Hormonal variables were measured in the same laboratory using commercially available kits as previously described^24^. Additionally, serum concentrations of sodium and potassium were measured. Every adrenal lesion was assessed with CT as per the following criteria: size, lateralization, tissue density measured in Hounsfield units (HU), and contrast washout values. CT can be performed with or without contrast enhancement. In our study, lesions with a density of ≤ 10 HU were considered benign. A tumour size > 5 cm is indicative of malignancy and is considered an indication for adrenalectomy^25^. In the adrenal mass, absolute washout is calculated in lesions with a density of > 10 HU, and it has been confirmed that a value > 60% is indicative of a benign lesion^12^. In our study, regular shape, size less than 5.0 cm, density ≤ 10 HU, absolute washout value > 60%, and relative washout > 40% were considered CT evidence of a benign adrenal mass. Abdominal CT was performed in all patients at the Radiology Department of our hospital. Moreover, every patient was screened for obesity, type 2 diabetes mellitus, impaired glucose tolerance (IGT), hyperlipidemia, nodular goiter, Hashimoto disease, Graves’ disease, heart failure, atrial fibrillation (AF), ischemic heart disease, renal failure, and hypertension, especially severe and resistant arterial hypertension, which was taken into consideration, defined according to World Health Organization (WHO) criteria. The data were extracted according to the criteria recommended by the Polish Society of Endocrinology for AI management^23^. All extracted data were complete and credible according to medical standards.

### Machine learning approach

**Figure 1:**
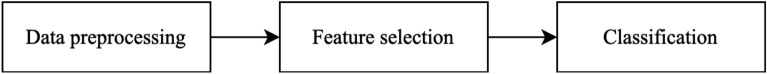
System stages.

In our study, we applied selected supervised ML methods with the main stages depicted in Fig. 1. In the preprocessing stage, nominal attributes were converted to numerical values using one-hot encoding, and all attributes were normalized to have the same range. During the experiments, we constructed a feature vector using all the available attributes and performed experiments with reduced feature sets selected using the backwards search method. The feature vectors were passed to the classification algorithm that assigned the subject to one of two classes: qualified or not qualified for adrenalectomy.

### Feature vector attributes

**Figure 2:**
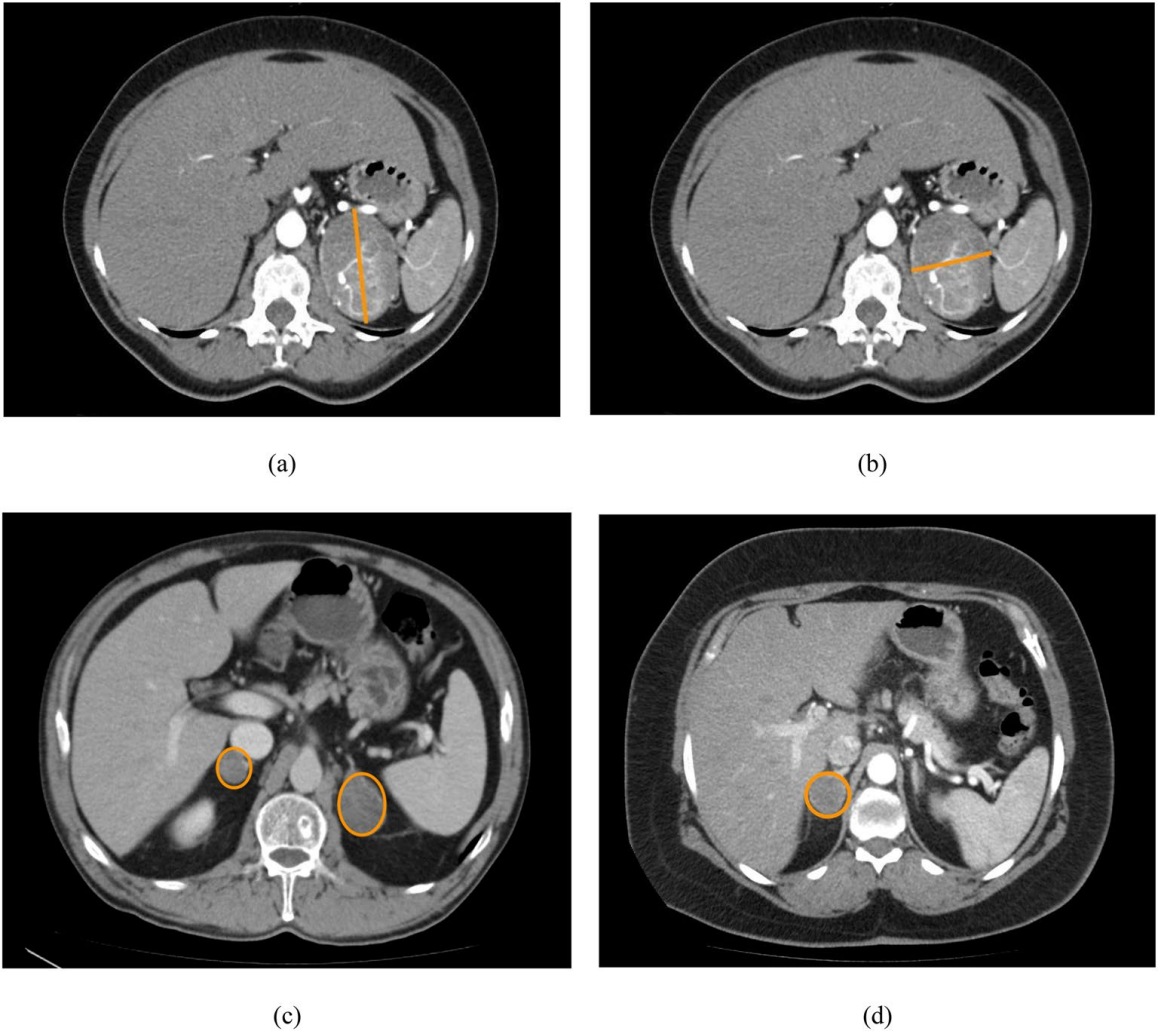
Examples CT image with adrenal tumour showing attributes used in this study: (**a**) maximal diameter for tumour with homogeneity feature absent and laterization feature present, (**b**) minimal diameter for tumour with homogeneity feature absent and laterization feature present, (**c**) tumour with laterization feature absent, (**d**) tumour with homogeneity feature present.

Each patient in the study had 24 attributes. Eight attributes represented measurements on a ratio scale, and the remaining 15 represented measurements on a nominal scale. All nominal attributes had two values: female/male for gender and the presence or absence of features for other attributes. Table 2 shows all attributes with their scales and the summary of their values. Examples of CT images depicting adrenal tumours, illustrating the attributes used in this study, were presented in Fig. 2. For the attributes on the quotient scale, the median and interquartile range were given. For the nominal attributes, the table contains the case counts for each of the two possible values.

**Table 2:** Attributes used to construct the feature vector. The summary column for attributes in the ratio scale contains the median and interquartile range (given in parentheses). For nominal attributes, the summary column contains the number of subjects within each of the two groups having specific attribute values: male (female) for gender and absent (present) for other nominal attributes.

Attribute	Scale	Summary
Gender	nominal	16 (17)
Age	ratio	57 (13)
Obesity	nominal	24 (9)
Hypertension	nominal	5 (28)
IGT	nominal	24 (9)
Hyperlipidemia	nominal	27 (6)
Type 2 diabetes mellitus	nominal	20 (13)
Thyroid nodules	nominal	11 (22)
Hashimoto disease	nominal	32 (1)
Graves disease	nominal	31 (2)
Heart failure	nominal	32 (1)
AF	nominal	28 (5)
Ischemic heart disease	nominal	30 (26)
Renal failure	nominal	24 (9)
Minimal diameter of the tumour	ratio	40 (26)
Maximum diameter of the tumour	ratio	34 (24)
Homogeneity	nominal	13 (20)
Lateralization	nominal	24 (9)
Serum sodium	ratio	138 (2)
Serum potassium	ratio	4 (1)
Suppression test with 1 mg of DXM	ratio	1.3 (1)
24 h urine collection for metanephrins	ratio	119 (133)
24 h urine collection for normetanephrins	ratio	548 (468)

### Classifiers

In our study, we used several classifiers, which are briefly described in this section^26,27^.

- Zero R—baseline approach that assigns examples to the majority class in the training set (ignores attribute values).
- One rule is a classifier that uses only a single attribute for classification and assigns the subject to the majority class with the same attribute value in the training set. If attribute selection is performed based on the accuracy measure, the selected attribute has the highest accuracy in the training set. The algorithm was applied to nominal attributes. The numerical attributes were converted to nominal values using the discretization procedure described in^28^ (with a minimum bucket size of 6).
- Naïve Bayes is a classifier based on Bayes’ theorem, with the assumption of feature independence. The probability that a given feature vector x belongs to class ${c}_{k}$ is given in Eq. (1).
  1 $$p(C={c}_{k}|X=x)=\frac{p({\text{x}}|C={c}_{k})*p(C={c}_{k})}{{\varvec{p}}({\varvec{X}}={\varvec{x}})}$$
  The predicted class $\widehat{{\text{c}}}$ can be selected using the maximum probability (MAP) rule (2).
  2 $$\widehat{c}=\underset{k\in 1\dots K}{\text{argmax}} p\left(C={c}_{k}|X=x\right)$$
  *K* is the number of classes.
- K-nearest neighbors—classifies the subject based on the plurality vote of its k-nearest neighbors, where the neighborship is assessed based on a distance measure applied to examples in the training set. In this study, we used the Euclidian distance.
- Logistic regression with ridge regularization models the probability that a given feature vector belongs to a particular class. It is based on the assumption that the logarithm of odds (log-odds) can be described using a linear combination of predictor variables, and thus, in case of two possible decision classes ($C={c}_{1}$ or $C={c}_{2}$), the probability of x having class $C={c}_{1}$ may be computed using formula (3).
  3 $$p\left(C={c}_{1}|X=x\right)=\frac{1}{1+{e}^{{-\beta }^{T}x}}$$
  The vector of the coefficients β is selected to minimize the cost function L (4).
  4 $$\begin{aligned} L & = - \mathop \sum \limits_{{i = 1}}^{N} L_{i} + r\beta ^{T} \beta ~, \\ L_{i} & = y_{i} ln\left( {p\left( {C = c_{1} {\text{|}}x_{i} } \right)} \right) + \left( {1 - y_{i} } \right)ln\left( {1 - p\left( {C = c_{1} {\text{|}}x_{i} } \right)} \right). \\ \end{aligned}$$
  where N is the number of examples in the training set, ${y}_{i}$ denotes whether sample *i* belongs to class ${c}_{1}$ (${y}_{i}=1$) or not (${y}_{i}=0$), ${x}_{i}$ is the feature vector of the i-th sample.
- The SVM classifier separates classes with a hyperplane that has the largest margin (distance to the nearest data point). In our case, we used a soft-margin SVM that allows data points to cross the hyperplane, thereby reducing the separation requirement. The soft-margin separating the hyperplane is determined by minimizing (5) under the constraints given by (6) and (7). The hyperplane is represented by vector w normal to the plane and scalar b. The value ${\xi }_{i}$ captures the margin violation for sample i. Scalar $\lambda$ is a regularization coefficient that controls the extent to which the margin violation is acceptable. There are N samples, where ${x}_{i}$ denotes the i-th sample feature vector and ${y}_{i}$ denotes the class of the sample (1 or − 1).
  5 $$\underset{w,b,{\xi }_{i}}{{\text{min}}}\frac{1}{2}{w}^{T}w+\lambda {\sum }_{i=1}^{N}{\xi }_{i}$$
  6 $${y}_{i}\left({w}^{T}{x}_{i}+b\right)\ge 1-{\xi }_{i},\mathrm{ i}\hspace{0.17em}=\hspace{0.17em}1\dots \mathrm{N }$$
  7 $${\xi }_{i}\ge 0,\mathrm{ i}\hspace{0.17em}=\hspace{0.17em}1\dots {\text{N}}$$
  To allow for nonlinear separation, the feature vectors ${x}_{i}$ can be transformed into another space, usually with more dimensions, where the hyperplane separation will result in nonlinear separation in the original space. The same effect is achieved by a kernel trick that computes the inner product in the transformed space without the explicit transformation of vectors from the original space. Popular types of kernels include linear, polynomial, and Gaussian radial basis function (RBF) kernels.
- C4.5 Decision Tree—classifier that generates a decision tree based on C4.5. The C4.5 algorithm uses entropy to measure information gain when selecting attributes to split during the tree creation process. The nodes of the tree represent the decision rules, and the leaves represent decisions. We used the J48 implementation of C4.5 in Weka.
- Random Forest—The algorithm creates a set of decision trees^29^, each learned using samples from the training set selected randomly with replacement and random subsets of features. The classification decision for a new sample is performed by voting—the decisions (votes) made by trees in the set are counted, and the class with the most votes wins. In this study, the set consisted of 100 trees.
- Artificial Neural Network—In this study, we used a feed-forward multilayer network with a sigmoid activation function in the hidden layers. The network was trained using stochastic gradient descent with momentum. The neural network consisted of three layers (input, hidden, and output), with the number of neurons in the hidden layer equal to the number of attributes and two neurons in the output layer (one for each class).
  All the variables in the equations in the manuscript are summarized in Table 3.

**Table 3:** Summary of the variables used in the equations presented in the manuscript.

Symbol	Meaning
*C*	Random variable representing the class of a sample
*K*	Number of classes
${c}_{k}$	The value of *C* for the sample of k-th class
*X*	Random variable representing the feature vector of a sample
*M*	Number of features
x	Feature vector representing a sample, $x={[1,{x}_{\left(1\right)},{x}_{,\left(2\right)},\dots ,{x}_{(M)}]}^{T}$
y	Scalar value representing the class of a sample
*K*	Number of classes
*p(A)*	Probability of event A
*p(A|B)*	Conditional probability of event A given event B has occured
$\beta$	Vector of coefficients in logistic regression, $\beta ={[1,{\beta }_{\left(1\right)},{\beta }_{\left(2\right)},\dots ,{\beta }_{\left(M\right)}]}^{T}$
*r*	Ridge regularization scalar coefficient in logistic regression
*w*	Normal vector defining SVM hyperplane, $={[{w}_{\left(1\right)},{w}_{\left(2\right)},\dots ,{w}_{\left(M\right)}]}^{T}$
${\xi }_{i}$	Scalar value controlling margin violation constraint in SVM for the i-th sample
$\lambda$	Regularization scalar coefficient in SVM

## Results

In this section, we present the results of the experiments conducted in this study. In all experiments, we used algorithms implemented in the Weka software package^30^.

### Experiment 1

During the first experiment, we evaluated 11 classifiers applied to the full attribute set, as shown in Table 4. The results were obtained using a tenfold stratified cross-validation scheme repeated 100 times with random reordering of the samples. Consequently, each classifier was trained and evaluated 1000 times on various datasets split into training (90%) and test (10%) subsets. Table 4 presents the average accuracy with standard deviations (SD) computed for the evaluations per classifier.

**Table 4:** Percent of properly classified subjects using all attributes.

Classifier	Accuracy (SD)
Zero R (baseline)	64,17 (7,50)
One rule	77.89 (24.55)
Naïve bayes	83.38 (19.02)
K-nearest neighbours (k = 1)	85.16 (18.56)
K-nearest neighbours (k = 3)	82.81 (19.87)
Logistic regression	77.96 (23.20)
Support vector machine (Linear)	90.98 (16.25)
Support vector machine (RBF)	64.17 (7.50)
C4.5 Decision tree	75.35 (22.38)
Random forest	84.24 (18.68)
Neural network	81.84 (20.65)

The number of patients qualified correctly and incorrectly were different. Therefore, the dataset was unbalanced with respect to the class attribute. Hence, the accuracy of the Zero-R classifier was determined to establish a baseline for further comparisons (Zero-R assigns the example to the most common class in the training set). Statistical analysis of the results performed with the paired t test, modified to account for using the same dataset multiple times with random reordering, proved that all methods except four (one rule, logistic regression, SVM with RBF kernel, C4.5 Decision Tree) were significantly better than the baseline (*p* < 0.05). As seen in Table 4, the best result of 91% was obtained for the SVM and linear kernel with soft margins. The K-nearest neighbors (with *k* = 1) gave the second-best result of 85%, followed by random forest with 84%. These results indicate that the application of ML methods may improve the decision-making process.

### Experiment 2

To evaluate the importance of attributes for classification accuracy, we applied the wrapper method with the backwards best-first search method, with search termination after five nonimproving nodes^31^. Attribute selection was performed on the training subset obtained from the cross-validation split. After the attribute selection, the classifier was trained and evaluated on the test subset of the cross-validation split. The procedure was performed using a tenfold cross-validation scheme and repeated five times with random reordering of the samples. Table 5 shows the percentage of times each attribute was selected; attributes that were selected more frequently were better (more stable) indicators for issuing correct decisions. The most frequently selected attributes were tumour homogeneity (100%), maximum tumour diameter (98%), and obesity (100%). For the classifier, we used an SVM with a linear kernel that gave the best results in Experiment 1.

**Table 5:** The percentage of times each attribute was selected using the wrapper method with a backwards search for SVM with a linear kernel in a tenfold cross-validation scheme.

Attribute	The precent of times each attribute was selected (%)
Gender	12
Age	42
Obesity	100
Hypertension	30
IGT	42
Hyperlipidemia	16
Type 2 diabetes	48
Thyroid nodules	34
Hashimoto disease	40
Graves disease	40
Heart failure	34
AF	28
Ischemic heart disease	56
Renal failure	68
Minimal diameter of the tumour	66
Maximum diameter of the tumour	98
Homogeneity	100
Lateralization	68
Serum sodium	76
Serum potassium	34
Suppression test with 1 mg of DXM	72
24 h urine collection for metanephrins	90
24 h urine collection for normetanephrins	94

### Experiment 3

In this experiment, we applied the attribute selection method from Experiment 2 combined with selected classifiers and evaluated the performance of the classifiers used on the reduced attribute set. The results were obtained with a tenfold cross-validation scheme repeated 100 times with random reordering of samples. The same classifier was used for attribute selection and classification processes. As seen in Table 6, prior attribute selections using the wrapper method did not lead to better accuracy of most trained classifiers; only in the case of K-nearest neighbors (*k* = 3) and C4.5 was a slight improvement observed.

**Table 6:** Percent of properly classified subjects with prior attribute selection.

Classifier with prior attribute selection	Accuracy (SD)
Naïve bayes	82.80(18.40)
Support vector machines (Linear)	84.76(19.57)
K-nearest neighbours (k = 1)	77.42(23.08)
K-nearest neighbours (k = 3)	83.93(19.55)
C4.5 Decision tree	78.69(21.08)
Random forest	80.34(20.02)

## Discussion

The decision to qualify a patient for surgery is not always correct, as verified by histopathological examination. In this study, correct qualification was confirmed in only 21 of the 33 selected patients. This highlights how significant problems with personalized medical approaches to the management of AI occur and delineates the need for improvement of diagnostic tools. We demonstrated the usefulness of ML predictive algorithms based on existing data for reliable automated and preoperative classification of AI. ML was found to enable a reasonable level of accuracy in qualifying patients for adrenalectomy. The results of this study seem to show that artificial intelligence can detect patterns that may help in making the correct decision. In developing our manuscript, we followed the requirements of providing the high quality and usefulness of our medical ML study^32^.

From the results of Experiment 1 in a group of people who met the criteria for surgery, ML methods produce promising results: 91% of correct decisions for SVM classifiers versus 64% correctness achieved by medical specialists. It should be mentioned that this is a preliminary study with a relatively small dataset. Enlarging the set allows the use of more complex classifiers, such as larger neural networks, that may lead to even better results. In this study, 23 attributes were used. Nevertheless, subsequent studies provide new diagnostic tools in patients with AI, e.g. the EURINE-ACT study presented a triple test with urine steroid metabolomics, imaging characteristics, and tumour diameter to improve the detection of ACC^33^. Hence, there are future perspectives to improve the application of ML techniques in the qualification for the surgical treatment of adrenal tumours through the involvement of more characteristics.

In Experiment 2, the attribute selection method was used to investigate the attributes that were most relevant to the correctness of the classification. The results obtained were consistent with expert knowledge: imaging features of the tumour, such as homogeneity and size, were found to be the most important. Additionally, 24-h urine collection for normetanephrins, 24-h urine collection for metanephrins, suppression test with 1 mg of DXM, and aldosterone/renin ratio were also indicated as very important factors. Interestingly, obesity is also important. In further investigation, in the case of decision trees, the obtained rule suggested that with a homogeneous tumour image, the patient’s obesity significantly increased the chance of a pathological lesion. However, an attempt to reduce the set of attributes in Experiment 3 using the selection method from Experiment 2 did not improve the classification accuracy. This may indicate that it is difficult to establish a simple rule using only a few factors that result in high decision accuracy and that most of the selected data may be relevant for decision-making.

In our work we performed tuning of classifier hyperparameters using linear and grid search methods with internal cross-validation split on the training set. However, probably due to limited size of our dataset, the search did not lead to significant improvement over default parameter values proposed by the authors of Weka software package. As the alternative, our future plans include application of swarm methods for hyperparameter tunning, and also for feature selection^34–36^.

This study has several limitations. One of them is the small sample size. Thus, validation of these results in a large and well-balanced study population is necessary before clinical application. A larger number of patients with histopathologically confirmed tumours would have improved the accuracy of our results. Another constraint is the retrospective nature of the study and its inherent limitations. Similar limitations have been repeatedly mentioned in studies presented in Table 1. The comparison of accuracy of our study with other studies is difficult because they have different designs and do not consider the same factors. In the case of our work, the best accuracy was obtained for the SVM classifier (90.98%) as an average of 1000 iterations of the learning process. It should be noted that the accuracy was determined on the test set, which was not used in the selection of features as well as not used in the learning process, therefore the presented accuracy values are unbiased estimators. In other studies, such as Yi’s research, there was no separation between the training and test sets^10^. Another important point to mention is that, in our study, selected ML techniques (including the best performing Linear SVM) achieved a statistically significant advantage in accuracy over patient qualification performed by medical personnel.

Nonetheless, a significant strength of our study lies in its pioneering nature. It is the first study to incorporate both imaging and hormonal test results in ML techniques, encompassing the full spectrum of lesions qualifying for surgical treatment. Despite its limitations, especially its limited accuracy, our study provides valuable insights that lay the groundwork for further research in this field. Future studies with larger and more diverse cohorts, along with prospective designs, are essential to validate and extend our findings for clinical application.

## Conclusions

ML-based methods could be used as an accurate diagnostic device to help avoid unnecessary surgeries in patients with benign and non-functional adrenal masses. However, our results have not been adopted in daily practice thus far, and further studies are needed to investigate the application of other attributes in the decision-making process and the extension of the training database.

## Data Availability

The datasets generated and analysed during the current study are available from the corresponding author on reasonable request.

## Abbreviations

AI: Adrenal incidentaloma
ACCs: Adrenocortical carcinomas
AF: Atrial fibrillation
ANN: Artificial neural network
AUC: Area under curve
CT: Computed tomography
DXM: Dexamethasone
HU: Hounsfield unit
IGT: Impaired glucose tolerance
LASSO: Least absolute shrinkage and selection operator
ML: Machine learning
MRI: Magnetic resonance imaging
ROC: Receiver operating characteristic
SD: Standard deviation
SVM: Support vector machine
WHO: World health organization

## References

[1] Hanna FWF (2022). Adrenal incidentaloma: Prevalence and referral patterns from routine practice in a large UK university teaching hospital. J Endocr Soc.

[2] Kebebew E (2021). Adrenal incidentaloma. N Engl J Med.

[3] Terzolo M (2011). AME position statement on adrenal incidentaloma. Eur J Endocrinol.

[4] Young WF (2007). Clinical practice. The incidentally discovered adrenal mass. N Engl J Med.

[5] Kloos RT, Gross MD, Francis IR, Korobkin M, Shapiro B (1995). Incidentally discovered adrenal masses. Endocr Rev.

[6] Fassnacht M (2016). Management of adrenal incidentalomas: European society of endocrinology clinical practice guideline in collaboration with the European network for the study of adrenal tumors. Eur J Endocrinol.

[7] Sung TY (2021). Factors associated with postoperative complications and costs for adrenalectomy in benign adrenal disorders. Surgery.

[8] Wielogorska M (2022). Application of machine learning techniques in a qualification for a surgical treatment of adrenal tumors. Eur. Congr. Endocrinol..

[9] Liu H (2022). Computed tomography-based machine learning differentiates adrenal pheochromocytoma from lipid-poor adenoma. Front Endocrinol (Lausanne).

[10] Yi X (2018). Adrenal incidentaloma: Machine learning-based quantitative texture analysis of unenhanced CT can effectively differentiate sPHEO from lipid-poor adrenal adenoma. J. Cancer.

[11] Maggio R (2022). Machine learning-based texture analysis in the characterization of cortisol secreting vs. non-secreting adrenocortical Incidentalomas in CT scan. Front. Endocrinol. (Lausanne).

[12] Moawad AW, Ahmed A, Fuentes DT, Hazle JD, Habra MA, Elsayes KM (2021). Machine learning-based texture analysis for differentiation of radiologically indeterminate small adrenal tumors on adrenal protocol CT scans. Abdom. Radiol. (NY).

[13] Elmohr MM (2019). Machine learning-based texture analysis for differentiation of large adrenal cortical tumours on CT. Clin Radiol.

[14] Yi X (2018). Radiomics improves efficiency for differentiating subclinical pheochromocytoma from lipid-poor adenoma: A predictive, preventive and personalized medical approach in adrenal incidentalomas. Epma j.

[15] Yang W (2023). Application of a radiomics machine learning model for differentiating aldosterone-producing adenoma from non-functioning adrenal adenoma. Bioengineering (Basel).

[16] Feliciani G (2024). Radiomics in the characterization of lipid-poor adrenal adenomas at unenhanced CT: Time to look beyond usual density metrics. Eur. Radiol..

[17] Zhang Z (2016). A gentle introduction to artificial neural networks. Ann. Transl. Med..

[18] Zou J, Han Y, So SS (2008). Overview of artificial neural networks. Methods Mol. Biol..

[19] Vrtačnik P (2018). Epigenetic enzymes influenced by oxidative stress and hypoxia mimetic in osteoblasts are differentially expressed in patients with osteoporosis and osteoarthritis. Sci. Rep..

[20] Deberneh HM, Kim I (2021). Prediction of type 2 diabetes based on machine learning algorithm. Int J Environ Res Public Health.

[21] Piskin FC (2023). A machine learning approach to distinguishing between non-functioning and autonomous cortisol secreting adrenal incidentaloma on magnetic resonance imaging using texture analysis. Ir. J. Med. Sci..

[22] Piskin FC, Akkus G, Yucel SP, Akbas B, Odabası F (2023). A radiomic signature based on magnetic resonance imaging to determine adrenal Cushing’s syndrome. Pol. J. Radiol..

[23] Bednarczuk T (2016). Adrenal incidentaloma in adults—management recommendations by the Polish Society of Endocrinology. Endokrynol. Pol..

[24] Adamska A (2022). Cardiovascular risk factors in mild adrenal autonomous cortisol secretion in a Caucasian population. Endocr. Connect..

[25] Thomasian NM, Kamel IR, Bai HX (2021). Machine intelligence in non-invasive endocrine cancer diagnostics. Nat. Rev. Endocrinol..

[26] Witten IH, Frank E, Hall MA, Pal CJ, Data M (2005). Practical machine learning tools and techniques.

[27] Trevor H, Robert T, Jerome F (2016). The elements of statistical learning: Data mining, inference, and prediction.

[28] Holte RC (1993). Very simple classification rules perform well on most commonly used datasets. Mach. Learn..

[29] Burrello J (2020). Development and validation of prediction models for subtype diagnosis of patients with primary aldosteronism. J. Clin. Endocrinol. Metab..

[30] Weka Data Mining Software. University of Waikato, New Zealand. https://www.cs.waikato.ac.nz/ml/weka/index.html. Accessed 25 Sep 2022

[31] Kohavi R, John GH (1997). Wrappers for feature subset selection. Artif. Intell..

[32] Cabitza F, Campagner A (2021). The need to separate the wheat from the chaff in medical informatics: Introducing a comprehensive checklist for the (self)-assessment of medical AI studies. Int. J. Med. Inform..

[33] Bancos I (2020). Urine steroid metabolomics for the differential diagnosis of adrenal incidentalomas in the EURINE-ACT study: A prospective test validation study. Lancet Diabetes Endocrinol..

[34] Yuan Y (2023). Coronavirus mask protection algorithm: A new bio-inspired optimization algorithm and its applications. J. Bionic. Eng..

[35] Yongliang Yuan Z, Ren J, Wang S, Wang Z, Xiaokai M, Wu (2022). Alpine skiing optimization: A new bio-inspired optimization algorithm. Adv. Eng. Softw..

[36] Shih-Wei Lin L, Ying KC, Chen SC, Zne-Jung (2008). Particle swarm optimization for parameter determination and feature selection of support vector machines. Exp. Syst. Appl..

